# Multi-Scale MRI, Radiology Reports, and Blood Biomarker–Guided Multimodal Deep Learning for Predicting Postoperative Recurrence in Cervical Cancer: A Multicenter Study

**DOI:** 10.3390/cancers18193075

**Published:** 2026-09-22

**Authors:** Yiyang Wu, Yao Ai, Yangyang Zhang, Long Zhang, Yifan Yang, Wenping Zhao, Ji Zhang, Yimou Fu, Jianping Wu, Yan Hu, Xiance Jin

**Affiliations:** 1 Department of Radiation and Medical Oncology, The First Affiliated Hospital of Wenzhou Medical University, Wenzhou 325000, China; 2 School of Basic Medical Science, Wenzhou Medical University, Wenzhou 325000, China; 3 Cixi Biomedical Research Institute, Wenzhou Medical University, Wenzhou 325000, China; 4 Department of Radiotherapy, The Second Affiliated Hospital & Yuying Children’s Hospital of Wenzhou Medical University, Wenzhou 325000, China; 5 Department of Radiotherapy, The Quzhou Affiliated Hospital of Wenzhou Medical University, Quzhou People’s Hospital, Quzhou 324000, China; 6 Department of Gynecology, The First Affiliated Hospital of Wenzhou Medical University, Wenzhou 325000, China

**Keywords:** cervical cancer, recurrence, multi-scale mode, MRI radiology report, deep learning

## Abstract

**Objectives**: Cervical cancer (CC) remains a leading cause of cancer-related morbidity and mortality among women worldwide, with postoperative recurrence posing a major challenge to long-term survival. Multimodal data integration, including imaging, textual, and clinical information, holds significant potential for enhancing prognostic prediction. This study aims to develop and validate a multimodal, multi-scale deep learning (DL) model for predicting recurrence in operable CC patients. **Methods**: This multicenter retrospective study included 445 operable CC patients with preoperative MRI and corresponding radiology reports from three institutions. A Multi-Scale Model (MSM) combining ConvNeXt and dual-path Vision Transformer (ViT) was developed. Textual features extracted from radiology reports using BERT were fused with imaging and clinical data to construct a multimodal network (MSM-TC). Model interpretability was enhanced using SHapley Additive exPlanations (SHAP) and attention visualization. **Results**: The MSM integrating ConvNeXt and ViT achieved receiver operating characteristic curve (AUC) values of 0.944, 0.837, and 0.681 in the training, internal validation, and external validation cohorts, respectively. Incorporating textual data (MSM-T) further improved performance (AUCs: 0.902, 0.860, and 0.742). The final multimodal model (MSM-TC) integrating imaging, textual, and clinical data demonstrated the highest predictive accuracy with AUCs of 0.930, 0.860, and 0.798 across training, internal validation, and external validation cohorts, respectively. Kaplan–Meier analysis confirmed that the model-derived risk score effectively stratified patients into high- and low-risk groups. **Conclusions**: Our multimodal, multi-scale DL framework robustly predicts recurrence and survival in operable CC patients, supporting its potential for individualized prognostic assessment and future clinical translation.

## Simple Summary

Cervical cancer is the fourth most common cancer among women worldwide. Although surgery provides effective treatment for patients with resectable disease, postoperative recurrence remains a major threat to long-term survival. Therefore, we aim to develop a multimodal model integrating preoperative imaging, unstructured radiology reports, and structured clinical information to predict long-term recurrence and survival after surgery. By using complementary information across multiple data modalities, this study may improve individualized risk stratification and provide the research community with a framework for multimodal prognostic modeling. Ultimately, the model could support personalized treatment decisions and risk-adapted follow-up strategies for patients with cervical cancer.

## 1. Introduction

Cervical cancer (CC) is the fourth most common malignancy in women worldwide, responsible for over 520,000 new cases and 260,000 deaths annually [1,2]. In early-stage disease, surgery is the mainstay of treatment, while chemoradiation is the most appropriate approach for locally advanced stages. However, a reported 30% postoperative recurrence rate in early-stage CC compromises long-term survival for patients with CC [3,4]. Clinical studies have demonstrated that additional postoperative adjuvant radiotherapy or chemotherapy may reduce recurrence [5,6], but these treatments are associated with increased toxicity and substantial socioeconomic burdens [7,8]. Identifying high-risk operable CC patients before therapy could guide treatment intensification while avoiding unnecessary toxicity in those with favorable prognoses.

Currently, the International Federation of Gynecology and Obstetrics (FIGO) clinical staging system serves as the primary tool for assessing recurrence risk and prognosis in patients with CC. However, significant prognostic heterogeneity exists among patients within the same FIGO stage [9]. Although nomograms incorporating factors such as lymph node status, tumor size, histology, and biomarkers can improve prediction [10], their performance remains limited because clinical factors insufficiently capture tumor biological heterogeneity [3,11,12].

With advances in artificial intelligence (AI), radiomics has enabled high-throughput extraction of imaging features for survival prediction in CC [13,14,15]. However, the robustness and generalizability of radiomics models are often limited by variability in imaging parameters across different studies. Recently, deep learning (DL) has been applied to improve prognostic predictions across cancer types by autonomously learning intricate nonlinear patterns directly from high-dimensional data [16]. Multimodal DL models further enhance prediction by integrating complementary information from different data sources [17]. For example, a DL-based multimodal pipeline integrating clinical characteristics, CT images, and laboratory test results has shown improved performance in diagnosing pulmonary infections [18]. In the specific context of CC, pelvic MRI is the standard imaging modality for staging and response assessment [8,19]. Meanwhile, medical imaging reports (MIRs)-rich in disease-related information documented by radiologists-are not routinely utilized in prognosis predictions due to their unstructured format [20]. The rise in Natural Language Processing (NLP) algorithms, such as Bidirectional Encoder Representations from Transformers (BERT), offers a promising solution to extract structured information from MIRs and facilitate clinical outcome research [21,22].

In this study, we developed a multi-scale, multimodal DL network that integrates MRI imaging features, MIRs data, and clinical information to improve the prediction of postoperative recurrence in patients with operable tumors. The robustness and clinical applicability of this model were further validated using both internal and external cohorts, demonstrating its reliability across diverse clinical settings.

## 2. Materials and Methods

### 2.1. Study Design

This retrospective multicenter study on postoperative recurrence prediction for CC was performed in four steps as shown in Supplementary Figure S1: (1) Imaging features were extracted from MRI using multi-scale feature extraction networks (ConvNeXt and Vision Transformer (ViT)) to capture hierarchical and heterogeneous radiographic characteristics. (2) Textual features were derived from MIRs using a BERT-based NLP model. (3) The extracted imaging features and textual features were integrated with relevant clinical parameters and subsequently input into a multilayer perceptron (MLP) to construct a predictive model. (4) The model-predicted score (risk score) was used for survival analysis, and further interpretability analysis was performed using SHapley Additive exPlanations (SHAP) to identify feature contributions.

### 2.2. Patients and Follow-Up

This retrospective study reviewed CC patients who underwent surgery from January 2008 to March 2021 across three hospitals. Inclusion criteria were as follows (Figure 1): (1) availability of both preoperative axial T2-weighted imaging (T2WI) MRI and corresponding MIRs within one month before surgery; (2) confirmed survival status within the first 5 years after surgery and adjuvant treatment (radiotherapy or concurrent chemoradiation). Exclusion criteria were: (1) absence of preoperative MRI; (2) history of other malignancies or concurrent malignancies; (3) poor image quality unsuitable for region-of-interest (ROI) delineation; (4) follow-up that was too short to ascertain the 5-year postoperative recurrence endpoint.

Patients were followed up every three months during the first two years after treatment, every six months for the subsequent three years, and annually thereafter. Recurrence occurring within 5 years after surgery was independently assessed by two experienced physicians based on imaging findings, pathological results, and clinical follow-up records. The primary endpoint of this study was postoperative recurrence within 5 years, whereas the secondary endpoint was disease-free survival (DFS). This study was approved by the institutional review board of all involved medical centers and conducted in accordance with the Declaration of Helsinki (ECRR no. 2019059), and the need for informed consent was waived due to its retrospective nature.

### 2.3. Images and Clinical Data

Preoperative MRI scans were obtained using a 3.0 T scanner (PHILIPS ACHIEVA, Philips Medical Systems Nederland B.V., Best, The Netherlands; GE Signa EXCITE and GE Signa HDxT, GE Healthcare, Waukesha, WI, USA) with a 16-channel phased-array body coil. The imaging parameters were as follows: field of view (FOV) = 220 × 220 mm, matrix = 336 × 336, thickness = 4 mm, and slice gap = 1 mm, using a fast spin-echo sequence. Target volumes were manually segmented as ROIs on axial T2WI by a radiologist with 8 years of experience, using 3D Slicer (version 5.6.2, 3D Slicer image computing platform|3D Slicer) [23]. Corresponding radiology reports were also collected to supplement the MRI findings. Clinical data were extracted from the electronic medical record system, including age, preoperative serum tumor markers such as squamous cell carcinoma antigen (SCC), cancer antigen 125 (CA125), and carbohydrate antigen 19-9 (CA19-9), as well as subsequent treatment choices regarding the administration of radiotherapy and/or chemotherapy. A clinical prediction model was developed using L1-regularized logistic regression based on the above parameters for subsequent comparative experiments. The details of the adjuvant treatments can be found in the Supplementary Methods S1.

### 2.4. Multi-Scale DL Network for Feature Extraction

A multi-scale network based on ConvNeXt and ViT was developed for DL feature extraction and fusion, with its overall architecture illustrated in Figure 2A [24]. The convolutional neural network (CNN) branch, constructed using ConvNeXt, consists of three sequential stages. Each stage employs a series of ConvNeXt Block Base modules to capture finer variations in tumor texture, margins, and internal heterogeneity. Before feature fusion, the outputs from different stages were projected into a unified feature space using 1 × 1 × 1 convolutions. The feature maps from all three stages were subsequently reshaped and concatenated along the depth dimension for downstream processing.

The ViT component adopts a dual-pathway multi-scale architecture comprising a Large Pathway (L-Pathway) and a Small Pathway (S-Pathway) for complementary feature representation [25]. The L-Pathway processes larger patches to provide a compact, coarse-scale representation of the overall tumor morphology and its spatial relationship with the surrounding cervical tissues. The S-Pathway uses smaller patches to generate a denser representation, enabling more sensitive modeling of spatial variations and heterogeneous patterns across the entire tumor region. Features from the two pathways are integrated to provide a comprehensive representation of cervical cancer across different spatial scales. Learnable positional embeddings are incorporated into both pathways to preserve spatial information during feature encoding.

Features from the ConvNeXt and dual-pathway ViT branches were integrated using a cross-attention mechanism [25]. The outputs of the L-Pathway and S-Pathway were first concatenated to form a unified multi-scale ViT representation. During cross-attention, the hierarchical features extracted by ConvNeXt were used as queries, whereas the fused ViT features served as keys and values. This design enables the local tumor structural information captured by the CNN branch to guide the selection and aggregation of spatially contextualized features from the ViT branches, thereby enhancing feature correspondence and complementarity across different spatial scales. A classification token and positional embeddings were subsequently incorporated into the fused feature sequence, which was further processed by a stack of Transformer encoder layers to achieve unified high-level representation learning for CC recurrence risk prediction and prognostic stratification.

### 2.5. Text Feature Extraction from Medical Reports

The textual branch was designed to transform unstructured radiology reports into quantitative feature vectors. In initial experiments, several baseline architectures incorporating BERT were evaluated, including BERT-Convolutional Neural Networks (BERT-CNN), BERT-Deep Pyramid Convolutional Neural Networks (BERT-DPCNN), BERT-Recurrent Neural Network (BERT-RNN), and BERT-Region-based Convolutional Neural Network (BERT-RCNN). Comparative analysis indicated that the standard BERT model [26,27] was most effective for this task, and it was therefore adopted as the core feature extractor. As illustrated in Figure 2B, the preprocessed text from each report was encoded by BERT to generate a 768-dimensional feature vector, capturing the essential semantic and contextual information pertinent to the imaging findings.

### 2.6. Multimodal Feature Fusion and Classification

The fusion of multimodal features was implemented using an MLP to model the complex, nonlinear relationships among the multimodal inputs (Figure 2D). Specifically, the BERT-derived text representation was concatenated with the selected clinical variables (Figure 2C) and imaging features to construct a unified multimodal representation for subsequent outcome prediction. This combined feature vector was then passed through a lightweight MLP head to generate the final prognostic prediction. This fusion architecture leverages the complementary strengths of each data modality—where imaging provides localized anatomical and textural details, text reports offer expert-derived semantic context, and clinical data supplies essential patient-specific factors. By integrating these complementary and information-rich features, the model obtains a more comprehensive representation of cervical cancer, thereby improving feature discriminability and robustness for recurrence prediction. The details regarding the model architecture and training procedure are provided in the Supplementary Methods S2.

### 2.7. Model Visualization and Interpretation

To enhance the clinical interpretability of the predictive model, multiple visualization techniques were employed. First, Gradient-weighted Class Activation Mapping (Grad-CAM) was used to generate heatmaps superimposed on the original MRI scans. These heatmaps highlight the anatomical regions that most strongly influenced the model’s prediction, providing insight into which image features—such as specific tumor characteristics or surrounding tissue—were deemed critical by the model. Secondly, the SHAP framework was employed to quantify and visualize the contribution of each input feature to the model’s output [28]. A scatter plot visualized the relationship between individual feature values and their corresponding SHAP values. This plot reveals the direction and magnitude of a feature’s effect across the cohort, illustrating both global trends and instance-level variations. Meanwhile, a bar chart ranked all input features by their mean absolute SHAP value, identifying the variables with the greatest overall influence on recurrence prediction.

### 2.8. Model Performance Evaluation and Statistical Analysis

Model discrimination for recurrence prediction was evaluated using the area under the receiver operating characteristic curve (AUC). Additional performance metrics, including accuracy (ACC), sensitivity, and specificity, were also reported. To assess the model’s prognostic stratification capability, the continuous risk score was dichotomized into high- and low-risk groups using an optimal cutoff determined by X-tile software (version 3.6.1) based on the training cohort. Kaplan–Meier curves were plotted for DFS, and the significance of the difference between risk groups was tested using the log-rank test (*p* < 0.05).

All statistical analyses were performed using R (version 4.3.0) (Supplementary Table S1) and Python (version 3.8). All deep learning models were implemented using the PyTorch (version 1.10.0) framework. Clinical and demographic characteristics were summarized using descriptive statistics. Differences between the recurrence and non-recurrence cohorts were assessed using the Chi-square test or Fisher’s exact test for categorical variables and the Mann–Whitney U test for continuous variables.

## 3. Results

### 3.1. Baseline Characteristics

A total of 445 consecutive CC patients were enrolled in this retrospective study. The cohort comprised 388 patients from Institution 1, who were randomly divided into a training set (*n* = 271) and an internal validation set (*n* = 117). An additional 57 patients from Institutions 2 and 3 served as an independent external validation cohort (Figure 1). The overall median age was 54 years (range 21–83 years). The median follow-up was 76 months (range 2–184 months). There were 75 patients with confirmed recurrence and 370 patients remained recurrence-free. The recurrence rate was 18.81%, 13.68%, and 14.04% in the training, internal validation, and external validation cohorts, respectively. Detailed clinicopathological characteristics of patients across all three cohorts are listed in Table 1.

### 3.2. Image- and Text-Based Model Performance

Table 2 and Figure 3 compare the performance of image-based and text-based DL models on the training, internal validation, and external validation datasets, evaluated using sensitivity, specificity, ACC, and AUC with 95% confidence intervals (CI). Among the image-based models, ConvNeXt demonstrated the most stable and competitive performance, achieving AUCs of 0.883 (95% CI: 0.825–0.935), 0.798 (95% CI: 0.623–0.939), and 0.656 (95% CI: 0.437–0.839) in the training, internal validation, and external validation cohorts, respectively. ViT also showed strong generalization, with AUCs of 0.766 and 0.704 on the internal and external validation sets, respectively. Among text-based models, BERT provided the most reliable overall performance with AUCs of 0.696 (0.568–0.830) and 0.681 (0.435–0.901) in the internal and external validation cohorts, respectively. Other architectures, such as BERT-CNN and BERT variants, showed strong performance on the training data but generalized inconsistently to the validation sets. ConvNeXt and ViT, along with BERT, provided the best balance between model fit and generalizability, supporting their selection for further comparative analysis in recurrence prediction.

### 3.3. Multi-Scale and Multi-Modal Models Comparison

Fusing the image features from ConvNeXt and ViT with the Multi-Scale Model (MSM) achieved AUCs of 0.944, 0.837, and 0.681 in the training, internal validation, and external validation, respectively. Adding text data improved the external validation from 0.681 (0.367–0.915) to 0.742 (0.549–0.913). Further integration of clinical data (MSM-TC) yielded the highest external validation AUC (0.798 (0.644–0.914)), demonstrating the best generalizability (Table 3, Figure 3). The no-information (NIR) was 0.863 and 0.860 in the internal and external validation cohorts, respectively (Supplementary Table S2). Subsequently, patients were stratified into high- and low-risk groups based on the risk score. Kaplan–Meier survival analysis revealed a statistically significant difference between groups (log-rank test *p* < 0.05), confirming that the risk score correlates with prognosis (Figure 4).

### 3.4. Ablation Studies of the MSM

To evaluate the contribution of each component in our Multi-Scale Model, we systematically removed key modules and assessed the impact on performance. As shown in Table 4, the complete model achieved the highest AUC in the internal validation cohort, with an AUC of 0.837. Removing ConvNeXt led to a marked decrease in internal validation performance, reducing the AUC to 0.709, suggesting that ConvNeXt provides important texture and contour information for robust feature extraction. Removing the L-Pathway and S-Pathway reduced the internal validation AUC to 0.717 and 0.770, respectively, indicating that both ViT pathways contributed to model performance, with the L-Pathway showing a larger impact on internal validation. In the external validation cohort, removing the S-Pathway resulted in the lowest AUC of 0.587, while removing ConvNeXt reduced the AUC to 0.635. These findings suggest that the S-Pathway may provide complementary fine-grained spatial information that is particularly important for external generalization, whereas ConvNeXt and the L-Pathway contribute to stable feature representation across cohorts.

### 3.5. Visualization Results

Figure 5 presents attention maps illustrating the focus of different models. For the CNN-based ConvNeXt model, the heatmap exhibits broad and diffuse activation across the cervical tumor and surrounding tissues, reflecting the tendency of convolutional kernels to capture locally continuous spatial patterns. In contrast, the ViT heatmap exhibits a more localized response concentrated within specific intratumoral regions. This pattern suggests that, by modeling global dependencies across image regions, the self-attention mechanism selectively emphasizes potentially discriminative morphological and structural features associated with cervical cancer recurrence. The MSM heatmap highlights the central tumor area while retaining moderate attention along the tumor margins and adjacent peritumoral tissue. This spatially balanced pattern suggests that MSM effectively integrates fine-grained intratumoral characteristics with broader tumor–microenvironment context, thereby providing a more comprehensive representation for cervical cancer recurrence prediction.

Figure 6 illustrates the feature importance of the final integrated model (MSM-TC) using SHAP values. The model-derived risk score was the most influential feature, confirming that the learned multi-modal representation effectively captures prognostic signal. This was followed in importance by clinical biomarkers such as CA125 and SCC, consistent with their established role in recurrence monitoring. Patient demographics (e.g., age) and treatment history also contributed, supporting the model’s ability to leverage multi-source data. An imaging-derived feature, Mean (ISHAF), was also included, underscoring the value of integrating MRI image information.

## 4. Discussion

Accurate prediction of recurrence in operable CC is crucial for guiding therapy and improving patient outcomes. In this study, we developed and validated a multimodal, multi-scale DL prognostic framework that integrates preoperative MRI, radiological text reports, and clinical data to predict recurrence risk. Our framework demonstrated robust performance, achieving AUCs of 0.860 and 0.798 in the internal and external validation cohorts, respectively.

The observed overall recurrence rate of 16.9% in our cohort aligns with the 10–20% range reported in the literature [3]. Traditional prognostic assessment in CC relies on clinical features such as age, stage, and serum markers [29,30]. Although PET/CT can detect recurrence in high-risk patients, current guidelines do not recommend its routine use because of high cost and limited evidence of improved outcomes. In line with prior studies, we found that SCC antigen levels, CA125 antigen levels, as well as FIGO stage were significantly associated with recurrence [31]. However, substantial outcome heterogeneity persists among patients with similar clinical characteristics, highlighting the need for more refined predictive tools.

DL has transformed medical image analysis, with CNNs and ViT emerging as powerful architectures [32]. For instance, Chen et al. developed and validated a DL-based pathological risk score integrating image and clinical data, achieving a C-index above 0.700 and an AUC of up to 0.800 for survival prediction in CC [33]. Similarly, Wang et al. developed a CNN model using CT images that attained a C-index of 0.81 for predicting survival in CC patients undergoing radiotherapy [34]. Growing evidence suggests that hybrid architectures combining CNNs and Transformers can better capture the hierarchical and global characteristics of medical images [35,36,37,38]. For example, Zhu et al. developed a hybrid CoTr model for CC MRI analysis, which achieved a C-index of 0.732 and effectively stratified patients into risk groups [39]. Building upon this paradigm, our proposed MSM further optimizes the fusion mechanism, achieving AUCs of 0.837 (internal) and 0.681 (external), outperforming single-branch models. Ablation analysis showed that removing ConvNeXt caused the greatest performance decline, suggesting that convolutional operations provided critical local feature extraction, particularly in limited-data settings. This supports prior evidence that CNNs can complement Transformers by enhancing local spatial modeling [40,41].

Accurate prognosis often requires the integration of multimodal data [42,43]. Most existing computational imaging tools focus on image-derived and structured clinical data, largely overlooking unstructured textural information. To address this gap, we incorporated radiology reports processed by BERT into the MSM framework, creating the multimodal MSM-T variant. This integration yielded a noticeable performance gain, raising the external validation AUC from 0.681 to 0.74. These findings suggest that textual semantics may provide complementary information and may improve model generalization, but this possibility requires confirmation in larger datasets with more recurrence events. Although the model accuracy was lower than the NIR, its relatively high sensitivity, specificity, and AUC suggest that it remained effective in identifying positive cases while maintaining good overall discriminative performance. (Supplementary Tables S2 and S3).

Interpretability remains a key challenge for DL in healthcare. Attention visualization showed that the MSM integrated complementary patterns from ConvNeXt and ViT, generating balanced heatmaps covering the tumor core and margins, which are clinically relevant to tumor heterogeneity, stromal invasion, and recurrence risk [44]. SHAP analysis further identified the model-derived risk score as the strongest predictor, followed by established biomarkers (CA125, SCC), demographic factors, and treatment history, ensuring predictions are both data-driven and clinically grounded. The proposed multimodal model not only outperforms single-modality baselines but also provides meaningful risk stratification (Figure 4), maintains interpretability through attention and SHAP analysis (Figure 5 and Figure 6), and demonstrates robust generalizability across cohorts (Table 3). These attributes support its potential clinical utility for personalized recurrence risk assessment.

This study has several limitations. First, variability in radiology reporting styles across institutions poses challenges for text-based generalization; future work will investigate more robust natural language processing strategies. Second, manual ROI delineation limits scalability; integrating automatic segmentation could enable an end-to-end workflow. Third, although external validation showed satisfactory discrimination, this cohort was relatively small, resulting in wide CIs and limited statistical precision. Therefore, the robustness and generalizability of the model remain uncertain, and larger multicenter validation is essential. Fourth, this study was not designed to determine the biological or treatment-related causes of recurrence. Treatment-related factors, including brachytherapy when applicable, were not evaluated and should be addressed in future studies. Finally, the current early-fusion approach could be extended by exploring intermediate or late-fusion strategies to potentially enhance performance.

## 5. Conclusions

We developed and validated a multimodal, multi-scale DL framework that integrates preoperative MRI, imaging reports, and clinical data to predict 5-year recurrence in operable CC patients. The model showed satisfactory discrimination in internal and external validation. By incorporating attention visualization and SHAP analysis, we enhanced model interpretability, facilitating its potential translation into clinical practice for personalized risk stratification and monitoring.

## Figures and Tables

**Figure 1:**
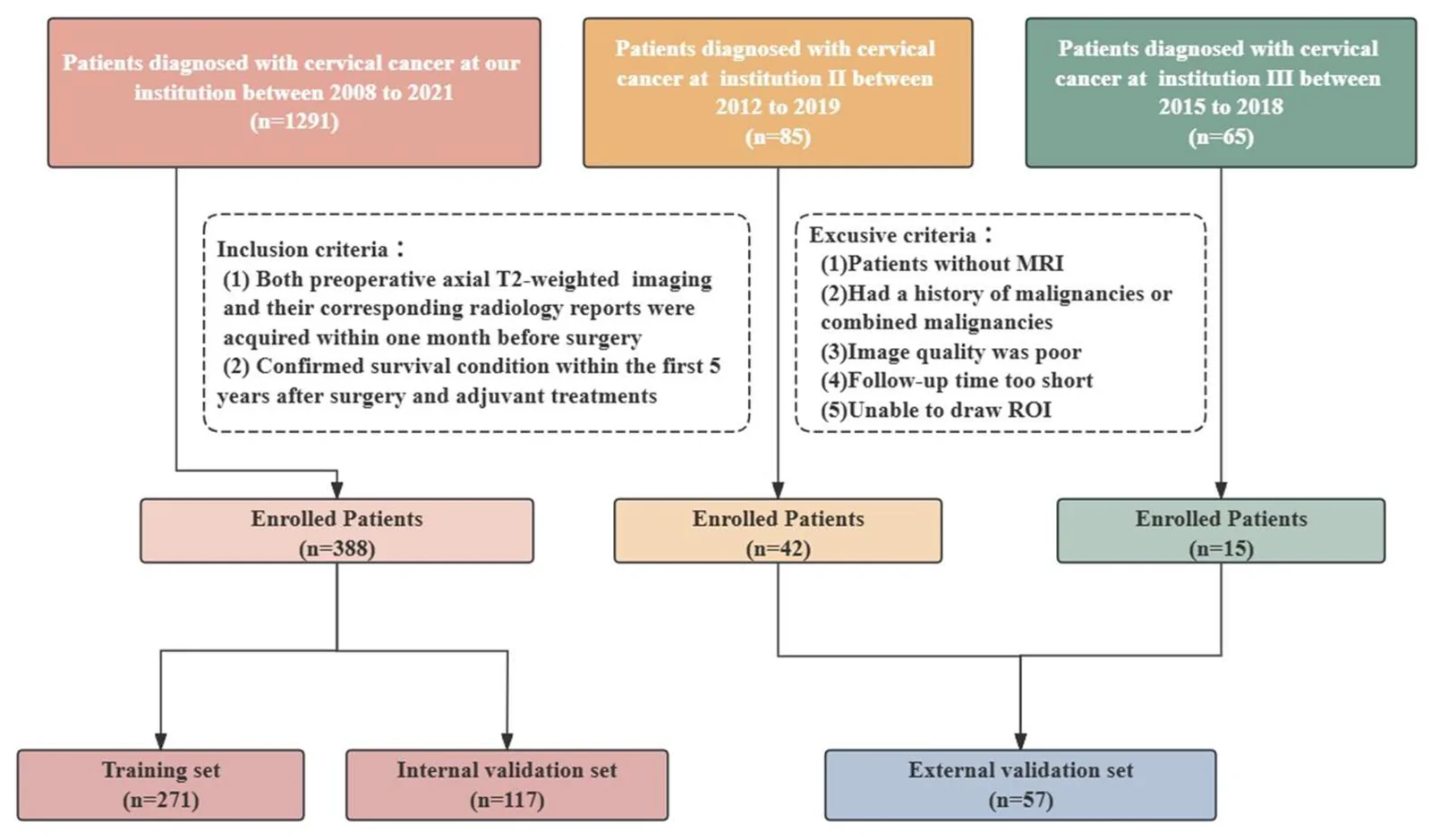
The flowchart of the case identification process.

**Figure 2:**
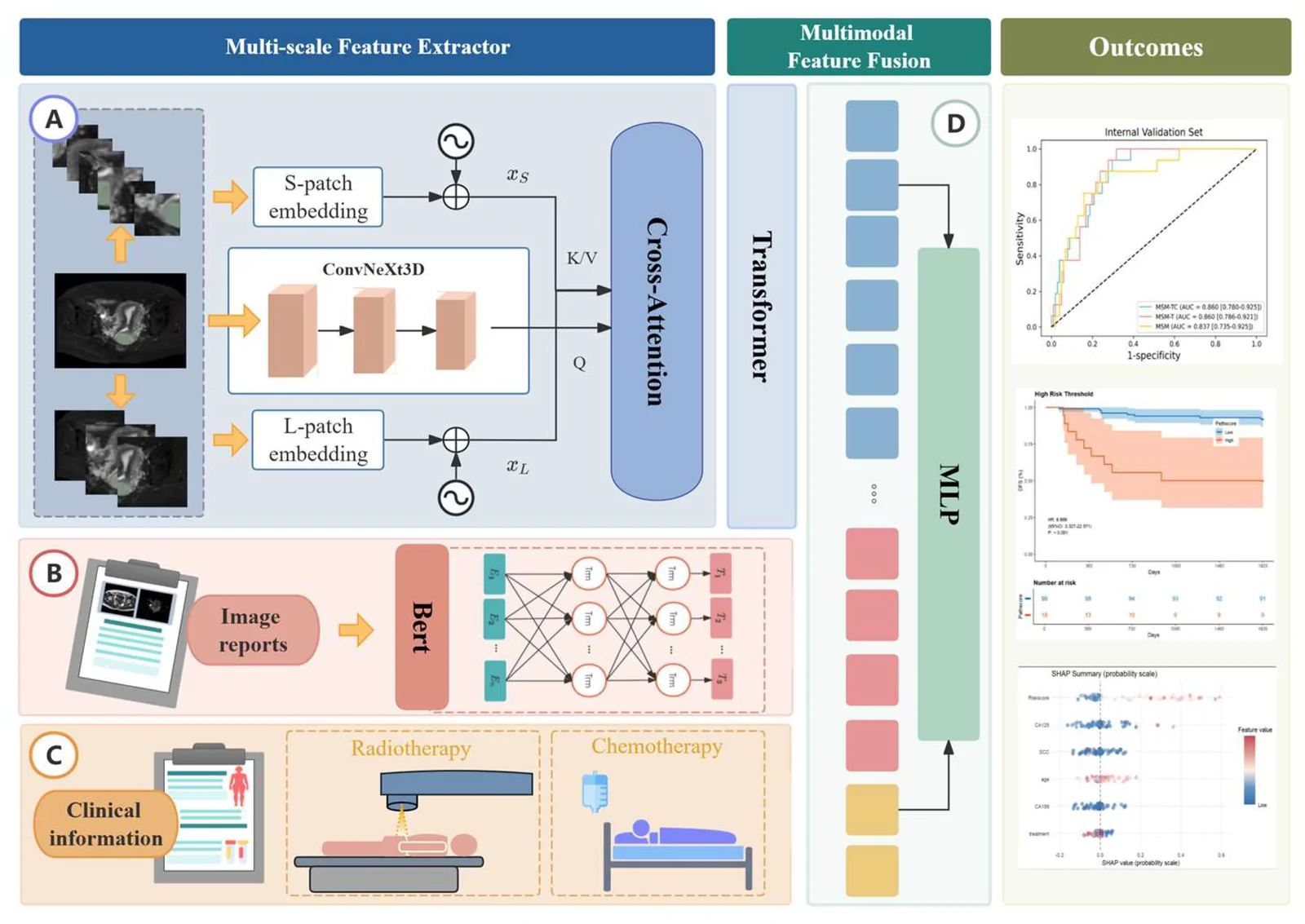
Workflow for model development. (**A**) Image feature extraction from MRI. (**B**) Text feature extraction from radiology reports. (**C**) Clinical feature extraction. (**D**) Multimodal feature fusion.

**Figure 3:**
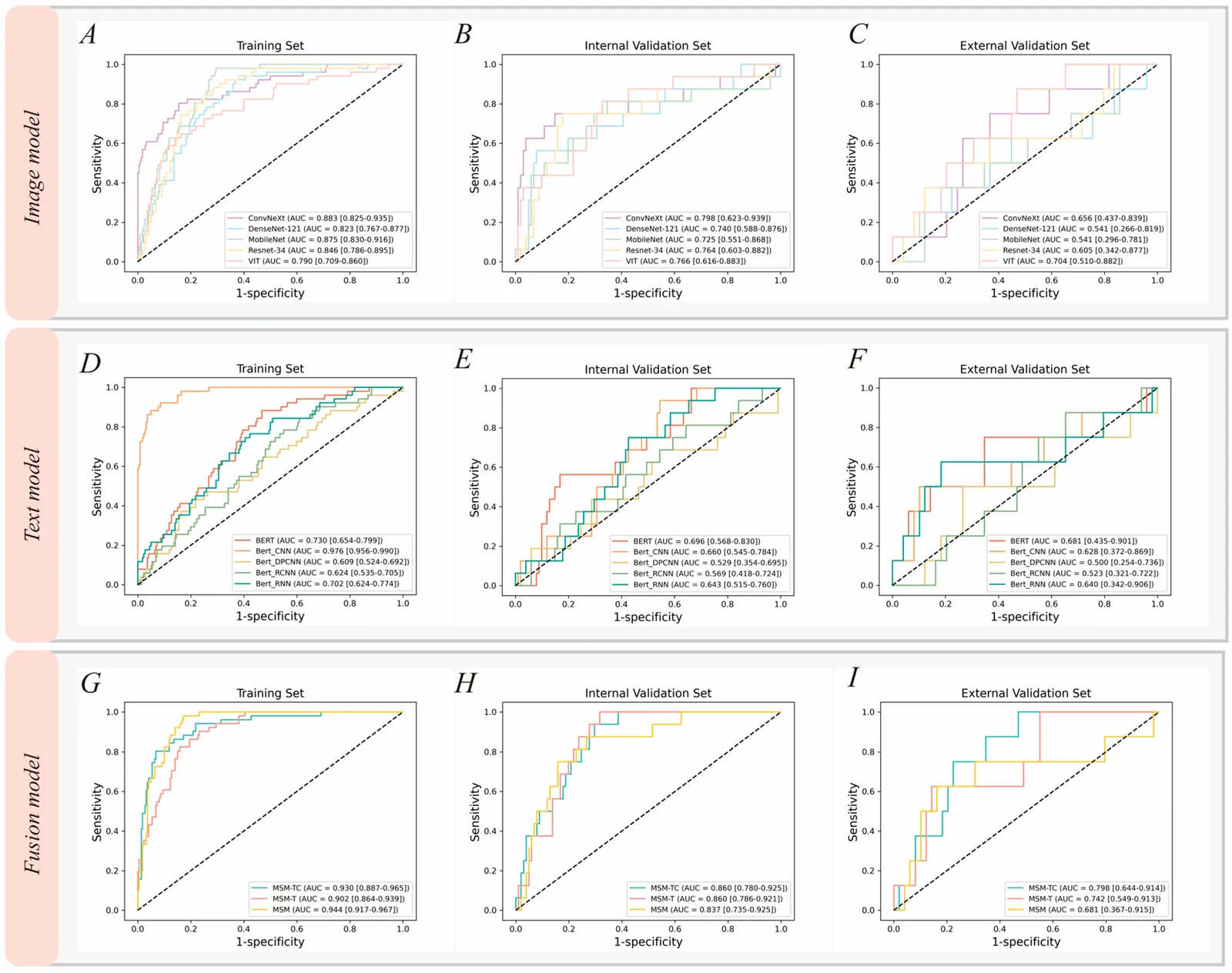
Performance of different architectures in predicting recurrence (**A**–**C**) Performance of five image-branch models on the training, internal validation, and external validation cohorts; (**D**–**F**) Performance of five text-branch models on the training, internal validation, and external validation cohorts; (**G**–**I**) Performance of the proposed fusion models, including the -scale model (MSM), the multimodal model incorporating text (MSM-T), and the final model integrating clinical features (MSM-TC) on the training, internal validation, and external validation cohorts (AUC: area under curve; CI: confidence interval).

**Figure 4:**
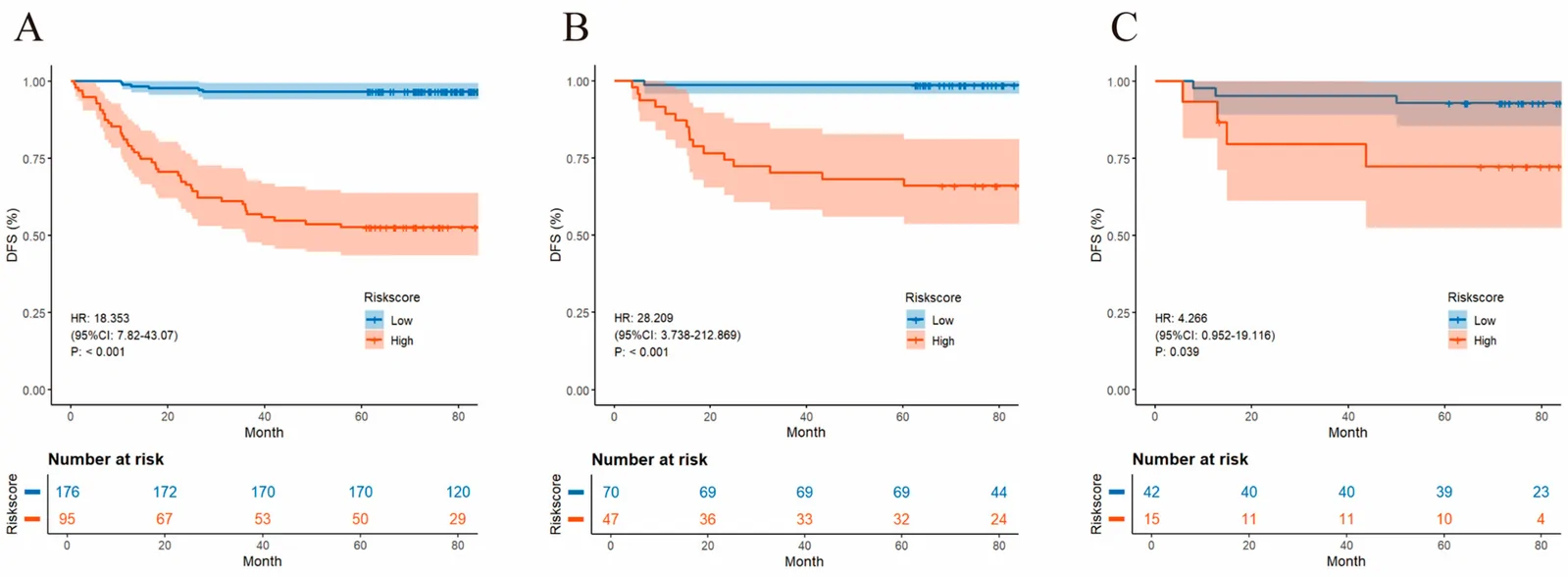
Model performance for predicting disease-free survival (DFS). (**A**) training cohort, (**B**) internal validation cohort, and (**C**) external validation cohort.

**Figure 5:**
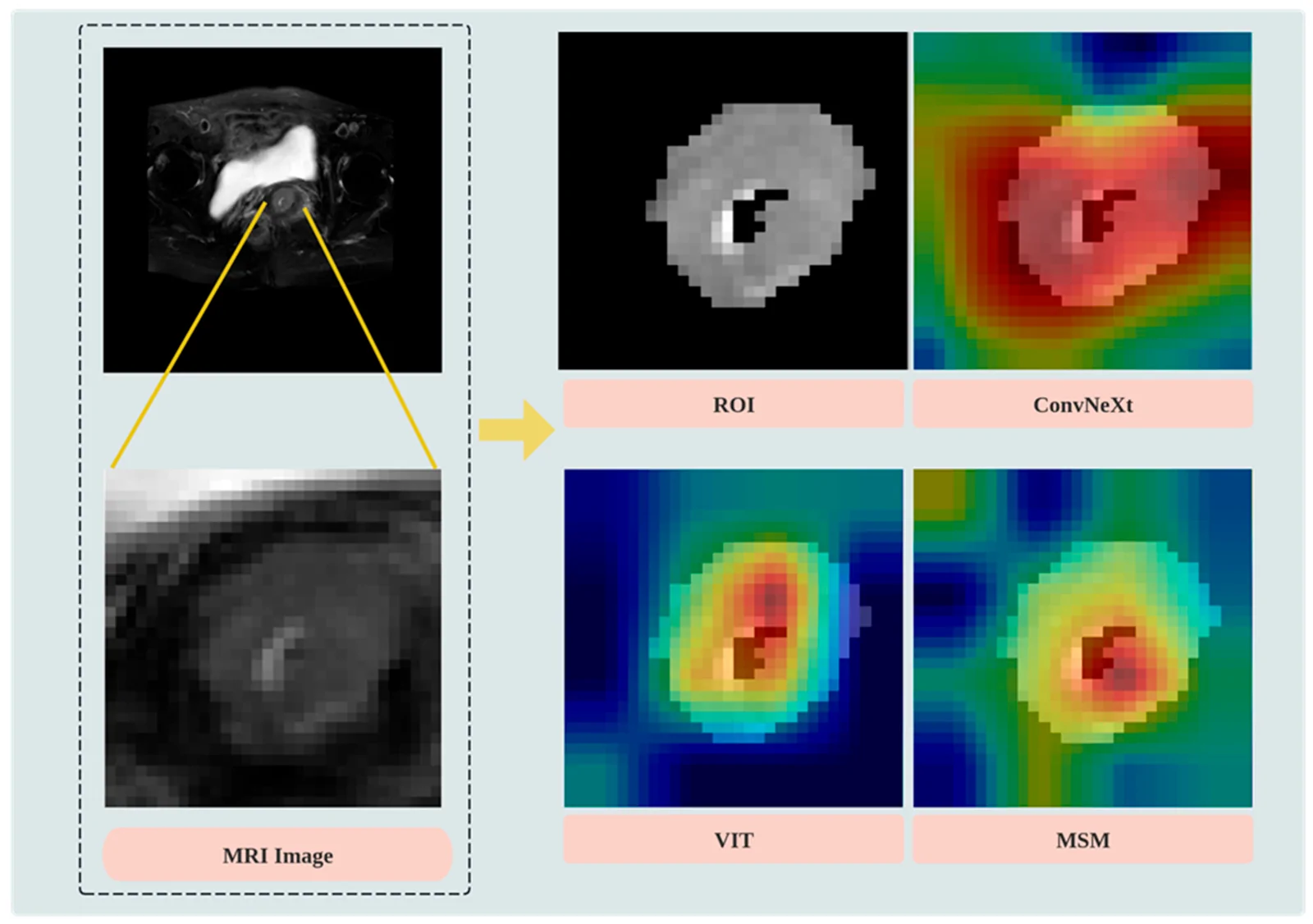
Comparison of attention maps across different models for lesion localization.

**Figure 6:**
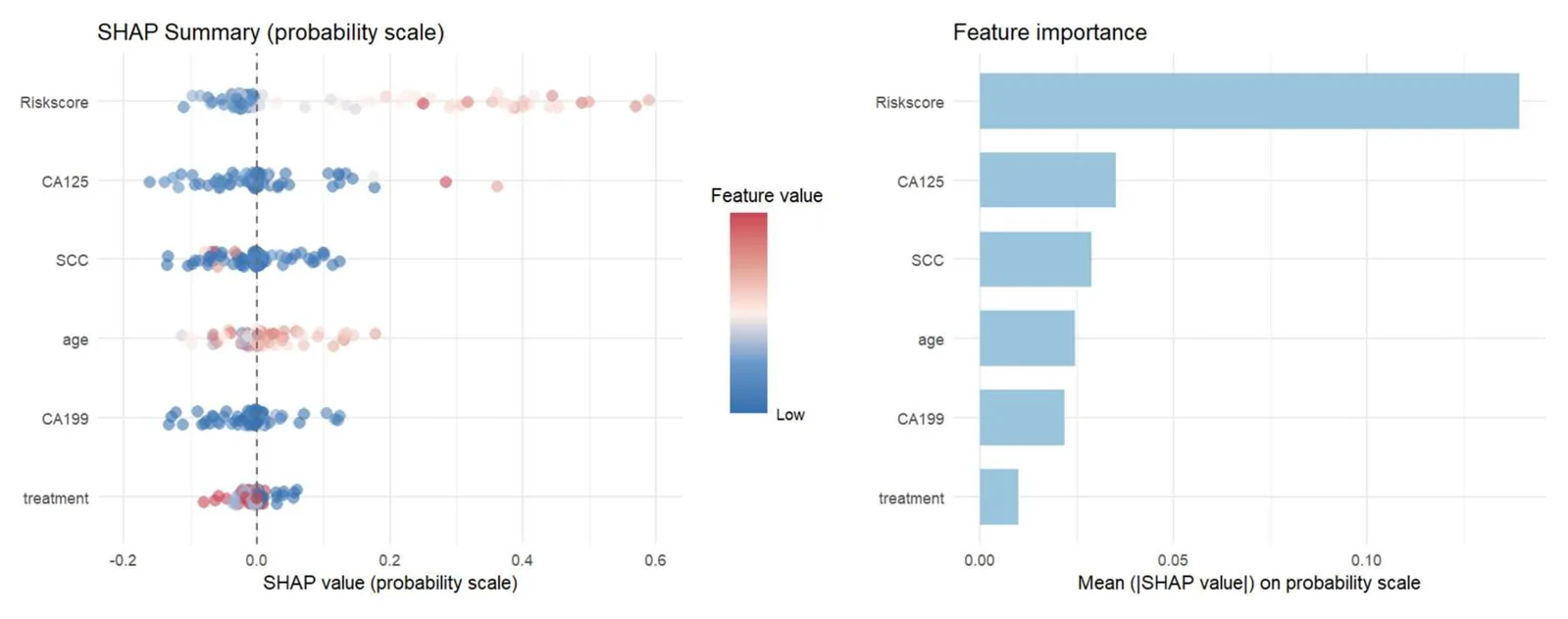
Predictive features for recurrence through Shapley Additive exPlanation (SHAP) value analysis.

**Table 1:** Clinical characteristics of patients in the training, internal validation, and external validation cohorts.

Characteristics	Training Cohort (*n* =271)	Internal Validation Cohort (*n* = 117)	External Validation Cohort (*n* = 57)	*p-*Value for Training vs. Internal Validation
Age(years, mean ± S)	53.44± 10.22	55.93± 9.64	51.82 ± 9.04	0.0257
Recurrence	51 (18.81%)	16 (13.68%)	8 (14.04%)	0.7349
Death	22 (8.12%)	10 (8.56%)	2 (3.51%)	0.5658
SCC-Ag (ng/mL)	2.32 ± 3.14	2.76 ± 4.13	3.46 ± 4.89	0.2569
CA19-9 (U/mL)	19.66 ± 24.50	20.49 ± 27.90	12.55 ± 10.22	0.7699
CA125 (U/mL)	21.82 ± 30.37	17.23 ± 16.15	28.78 ± 42.65	0.1242
Squamous cell carcinoma	234	108	53	0.123
Non-squamous cell carcinoma	37	9	4	
FIGO Stage				0.6495
IA	2	2	3	
IB	144	61	34	
IIA	110	48	17	
IIB	1	2	0	
IIIC	14	4	3	
Postoperative treatment				0.9608
Radiotherapy	52	29	11	
Chemotherapy	38	11	2	
Chemoradiotherapy	75	43	14	
Without Chemoradiotherapy	106	34	30	

Abbreviations: SCC-Ag: Squamous cell carcinoma; FIGO: International Federation of Gynecology and Obstetrics; CA19-9: carbohydrate antigen 19-9; CA125: Cancer antigen 125; *p*-value is calculated from the univariate association test between subgroups. χ 2 test and Fisher’s exact test for categorized variables; two-sample t-test for continuous variables.

**Table 2:** Performance of different architectures in predicting recurrence.

	Model	Dataset	Sensitivity (95% CI)	Specificity (95% CI)	ACC (95% CI)	AUC (95% CI)
Image model	ResNet-34	Training	0.882 (0.766–0.945)	0.713 (0.650–0.769)	0.745 (0.690–0.794)	0.846 (0.786–0.895)
		Internal Validation	0.750 (0.505–0.898)	0.822 (0.736–0.884)	0.812 (0.732–0.872)	0.764 (0.603–0.882)
		External Validation	0.500 (0.215–0.785)	0.796 (0.664–0.885)	0.754 (0.629–0.848)	0.605 (0.342–0.877)
	MobileNet	Training	0.980 (0.897–0.997)	0.705 (0.641–0.761)	0.757 (0.702–0.804)	0.875 (0.830–0.916)
		Internal Validation	0.812 (0.570–0.934)	0.654 (0.557–0.739)	0.675 (0.586–0.753)	0.725 (0.551–0.868)
		External Validation	0.375 (0.137–0.694)	0.816 (0.686–0.900)	0.754 (0.629–0.848)	0.541 (0.296–0.781)
	DenseNet-121	Training	0.922 (0.815–0.969)	0.631 (0.566–0.693)	0.686 (0.629–0.739)	0.823 (0.767–0.877)
		Internal Validation	0.563 (0.332–0.769)	0.921 (0.852–0.959)	0.872 (0.799–0.921)	0.740 (0.588–0.876)
		External Validation	0.625 (0.306–0.863)	0.551 (0.413–0.682)	0.561 (0.433–0.682)	0.541 (0.266–0.819)
	VIT	Training	0.628 (0.490–0.747)	0.859 (0.807–0.899)	0.816 (0.765–0.857)	0.790 (0.709–0.860)
		Internal Validation	0.813 (0.570–0.934)	0.673 (0.577–0.757)	0.692 (0.604–0.769)	0.766 (0.616–0.883)
		External Validation	0.875 (0.529–0.978)	0.531 (0.394–0.663)	0.579 (0.450–0.698)	0.704 (0.510–0.882)
	ConvNeXt	Training	0.804 (0.675–0.890)	0.845 (0.792–0.887)	0.838 (0.789–0.877)	0.883 (0.825–0.935)
		Internal Validation	0.750 (0.505–0.898)	0.852 (0.769–0.908)	0.838 (0.760–0.894)	0.798 (0.623–0.939)
		External Validation	0.750 (0.409–0.929)	0.633 (0.493–0.753)	0.649 (0.519–0.760)	0.656 (0.437–0.839)
Text model	BERT	Training	0.882 (0.766–0.945)	0.532 (0.466–0.597)	0.598 (0.538–0.654)	0.730 (0.654–0.799)
		Internal Validation	0.563 (0.332–0.769)	0.831 (0.747–0.892)	0.795 (0.713–0.858)	0.696 (0.568–0.830)
		External Validation	0.750 (0.409–0.929)	0.653 (0.513–0.771)	0.668 (0.537–0.775)	0.681 (0.435–0.901)
	BERT-CNN	Training	0.922 (0.815–0.969)	0.914 (0.869–0.944)	0.915 (0.876–0.943)	0.976 (0.956–0.990)
		Internal Validation	0.938 (0.717–0.989)	0.455 (0.362–0.552)	0.521 (0.431–0.610)	0.660 (0.545–0.784)
		External Validation	0.500 (0.215–0.785)	0.898 (0.782–0.956)	0.842 (0.726–0.915)	0.628 (0.372–0.869)
	BERT-DPCNN	Training	0.431 (0.305–0.567)	0.782 (0.723–0.831)	0.716 (0.659–0.766)	0.609 (0.524–0.692)
		Internal Validation	0.437 (0.231–0.668)	0.713 (0.618–0.792)	0.675 (0.586–0.753)	0.529 (0.354–0.695)
		External Validation	0.500 (0.215–0.785)	0.735 (0.597–0.838)	0.702 (0.573–0.805)	0.500 (0.254–0.736)
	BERT-RNN	Training	0.765 (0.632–0.860)	0.577 (0.511–0.641)	0.613 (0.553–0.669)	0.702 (0.624–0.774)
		Internal Validation	0.750 (5050–8982)	0.574 (0.477–0.666)	0.598 (0.508–0.683)	0.643 (0.515–0.760)
		External Validation	0.625 (0.306–0.863)	0.816 (0.686–0.900)	0.789 (0.667–0.875)	0.640 (0.342–0.906)
	BERT-RCNN	Training	0.784 (0.654–0.875)	0.450 (0.386–0.516)	0.513 (0.454–0.572)	0.624 (0.535–0.705)
		Internal Validation	0.812 (0.570–0.934)	0.356 (0.267–0.454)	0.419 (0.333–0.509)	0.569 (0.418–0.724)
		External Validation	0.875 (0.529–0.977)	0.347 (0.229–0.487)	0.421 (0.302–0.550)	0.523 (0.321–0.722)
Clinical Model		Training	0.333 (0.204–0.468)	0.873 (0.825–0.916)	0.771 (0.720–0.819)	0.582 (0.485–0.674)
		Internal Validation	0.312 (0.083–0.545)	0.861 (0.798–0.926)	0.786 (0.709–0.863)	0.641 (0.516–0.764)
		External Validation	0.111 (0.000–0.375)	0.880 (0.805–0.942)	0.804 (0.717–0.880)	0.675 (0.525–0.818)

Abbreviations: AUC, area under the curve; ACC, accuracy; CI, confidence interval.

**Table 3:** Performance of multi-scale and Multi-Modal Models in predicting recurrence.

Model	Dataset	Sensitivity (95% CI)	Specificity (95% CI)	ACC (95% CI)	AUC (95% CI)
MSM	Training	0.980 (0.897–0.997)	0.827 (0.772–0.872)	0.856 (0.809–0.893)	0.944 (0.917–0.967)
	Internal Validation	0.875 (0.640–0.965)	0.733 (0.639–0.809)	0.752 (0.667–0.822)	0.837 (0.735–0.925)
	External Validation	0.625 (0.306–0.863)	0.837 (0.710–0.915)	0.807 (0.687–0.889)	0.681 (0.367–0.915)
MSM-T	Training	0.902 (0.790–0.957)	0.768 (0.708–0.819)	0.793 (0.741–0.837)	0.902 (0.864–0.939)
	Internal Validation	1.000 (0.806–1.000)	0.683 (0.587–0.766)	0.727 (0.639–0.799)	0.860 (0.786–0.921)
	External Validation	0.625 (0.306–0.863)	0.857 (0.733–0.929)	0.825 (0.706–0.902)	0.742 (0.549–0.913)
MSM-TC	Training	0.804 (0.675–0.890)	0.932 (0.891–0.958)	0.908 (0.867–0.937)	0.930 (0.887–0.965)
	Internal Validation	0.938 (0.717–0.989)	0.703 (0.608–0.783)	0.735 (0.649–0.807)	0.860 (0.780–0.925)
	External Validation	0.750 (0.409–0.928)	0.776 (0.641–0.870)	0.772 (0.648–0.862)	0.798 (0.644–0.914)

Abbreviations: MSM: Multi-Scale Model; MSM-T: Multi-Scale Model with Text; MSM-TC: Multi-Scale Model with text and clinical data.

**Table 4:** The results of ablation studies.

Architecture			Internal Validation		External Validation	
ConvNeXt	L-Pathway	S-Pathway	AUC	95%CI	AUC	95%CI
	√	√	0.709	0.558–0.842	0.635	0.415–0.837
√		√	0.717	0.600–0.825	0.691	0.484–0.870
√	√		0.770	0.640–0.881	0.587	0.362–0.806
√	√	√	0.837	0.735–0.925	0.681	0.367–0.915

Abbreviations: L-Pathway: large pathway; S-Pathway: small pathway.

## Data Availability

The datasets generated and analyzed during the current study are not publicly available due to privacy concerns but are available from the corresponding author upon reasonable request.

## Supplementary Materials

The following supporting information can be downloaded at: https://www.mdpi.com/article/10.3390/cancers18193075/s1, Figure S1: The study design and analytical pipeline; Table S1: R packages; Table S2: Performance of different architectures in predicting recurrence; Methods S1: Adjuvant treatments; Table S3: Performance of Multi-Scale and Multi-Modal Models in predicting recurrence; Methods S2: Preprocessing, training methodology and model architecture.

## Abbreviations

The following abbreviations are used in this manuscript:

CC	Cervical cancer
MRI	Magnetic resonance imaging
T2WI	T2-weighted imaging
AI	Artificial intelligence
DL	Deep learning
CNN	Convolutional neural network
VIT	Vision Transformer
MLP	Multilayer perceptron
SHAP	SHapley Additive exPlanations
FIGO	International Federation of Gynecology and Obstetrics
ROC	Receiver operating characteristic
CI	Confidence interval
SCC	Carcinoma antigen
CA125	Cancer antigen 125
CA19-9	Cancer antigen 19-9
AUC	Area under the curve
ACC	Accuracy
DFS	Disease-free survival
ROI	Region-of-interest
L-Pathway	Large Pathway
S-Pathway	Small Pathway
BERT-CNN	BERT-Convolutional Neural Networks
BERT-DPCNN	BERT-Deep Pyramid Convolutional Neural Networks
BERT-RNN	BERT-Recurrent Neural Network
BERT-RCNN	BERT-Region-based Convolutional Neural Network
Grad-CAM	Gradient-weighted Class Activation Mapping
MSM	Multi-Scale Model
MSM-T	Multimodal model incorporating text
MSM-TC	The final model integrating clinical features
