# Supplementary material for: Multi-Scale MRI, Radiology Reports, and Blood Biomarker–Guided Multimodal Deep Learning for Predicting Postoperative Recurrence in Cervical Cancer: A Multicenter Study
Source: Cancers (Basel). 2026 Sep 22;18(19):3075. doi: 10.3390/cancers18193075 (PMC13664258; doi:10.3390/cancers18193075)
Supplement: Supplementary file 1 [file cancers-18-03075-s001.zip › cancers-4540704-supplementary.pdf]

Supplementary Materials

1. Figure S1: The study design and analytical pipeline.……………..……..…... 2
2. Table S1: R packages………………………..…………………………...……3
3. Table S2………………..………..…………………………….……………….4
4. Table S3...……………………………………………………………………….5
5. Methods S1: Adjuvant treatments………………………………………………8
6. Methods S2: Preprocessing, training methodology and model architecture……9

Figure S1: The study design and analytical pipeline.

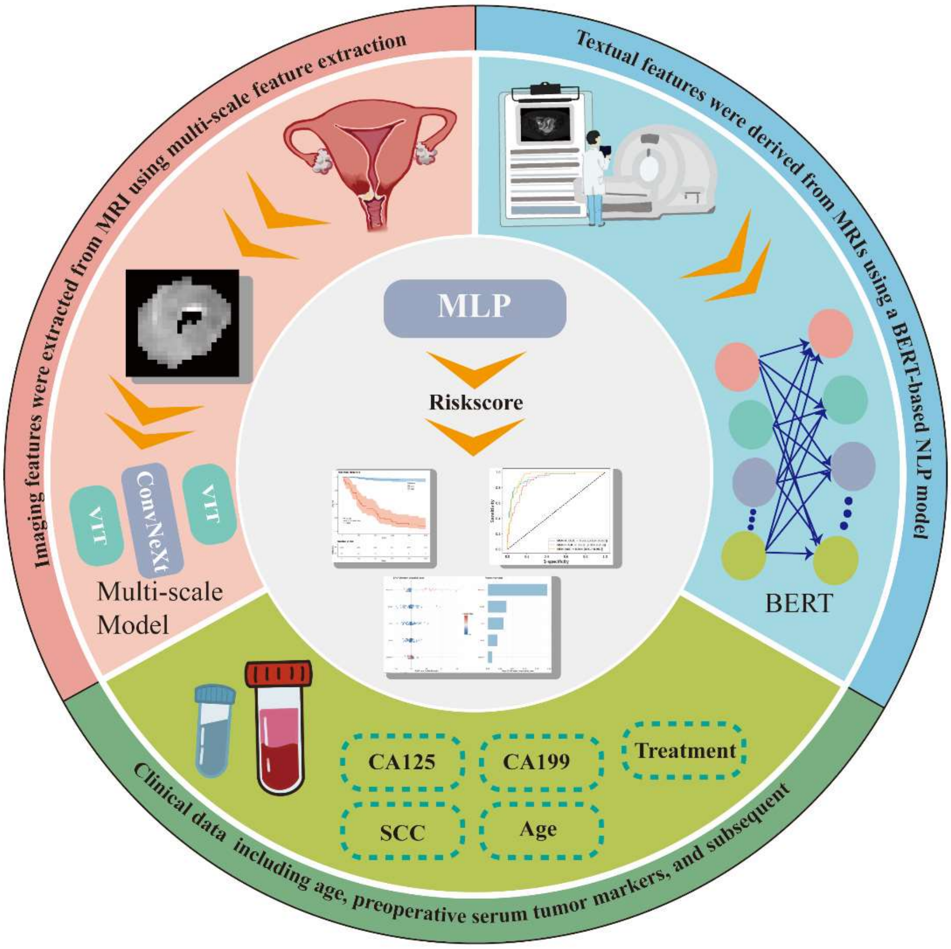

Table S1: R packages

Numbers	Name
1	ggplot2
2	pROC
3	readxl
4	glmnet
5	ggplot2
6	tidyr
7	xgboost
8	dplyr
9	scales
10	readxl
11	survival
12	survminer

Table S2 Performance of different architectures in predicting recurrence

	Model	Dataset	Sensitivity (95% CI)	Specificity (95% CI)	ACC (95% CI)	NIR	AUC (95% CI)
Image model	ResNet-34	Training	0.882 (0.766-0.945)	0.713 (0.650-0.769)	0.745 (0.690-0.794)	0.812	0.846 (0.786-0.895)
		Internal Validation	0.750 (0.505-0.898)	0.822 (0.736-0.884)	0.812 (0.732-0.872)	0.863	0.764 (0.603-0.882)
		External Validation	0.500 (0.215-0.785)	0.796 (0.664-0.885)	0.754 (0.629-0.848)	0.860	0.605 (0.342-0.877)
	MobileNet	Training	0.980 (0.897-0.997)	0.705 (0.641-0.761)	0.757 (0.702-0.804)	0.812	0.875 (0.830-0.916)
		Internal Validation	0.812 (0.570-0.934)	0.654 (0.557-0.739)	0.675 (0.586-0.753)	0.863	0.725 (0.551-0.868)
		External Validation	0.375 (0.137-0.694)	0.816 (0.686-0.900)	0.754 (0.629-0.848)	0.860	0.541 (0.296-0.781)
	DenseNet-121	Training	0.922 (0.815-0.969)	0.631 (0.566-0.693)	0.686 (0.629-0.739)	0.812	0.823 (0.767-0.877)
		Internal Validation	0.563 (0.332-0.769)	0.921 (0.852-0.959)	0.872 (0.799-0.921)	0.863	0.740 (0.588-0.876)
		External Validation	0.625 (0.306-0.863)	0.551 (0.413-0.682)	0.561 (0.433-0.682)	0.860	0.541 (0.266-0.819)
	VIT	Training	0.628 (0.490-0.747)	0.859 (0.807-0.899)	0.816 (0.765-0.857)	0.812	0.790 (0.709-0.860)
		Internal Validation	0.813 (0.570-0.934)	0.673 (0.577-0.757)	0.692 (0.604-0.769)	0.863	0.766 (0.616-0.883)
		External Validation	0.875 (0.529-0.978)	0.531 (0.394-0.663)	0.579 (0.450-0.698)	0.860	0.704 (0.510-0.882)
	ConvNeXt	Training	0.804 (0.675-0.890)	0.845 (0.792-0.887)	0.838 (0.789-0.877)	0.812	0.883 (0.825-0.935)
		Internal Validation	0.750 (0.505-0.898)	0.852 (0.769-0.908)	0.838 (0.760-0.894)	0.863	0.798 (0.623-0.939)
		External Validation	0.750 (0.409-0.929)	0.633 (0.493-0.753)	0.649 (0.519-0.760)	0.860	0.656 (0.437-0.839)
Text model	BERT	Training	0.882 (0.766-0.945)	0.532 (0.466-0.597)	0.598 (0.538-0.654)	0.812	0.730 (0.654-0.799)

Model	Dataset	Sensitivity (95% CI)	Specificity (95% CI)	ACC (95% CI)	NIR	AUC (95% CI)
	Internal Validation	0.563 (0.332-0.769)	0.831 (0.747-0.892)	0.795 (0.713-0.858)	0.863	0.696 (0.568-0.830)
	External Validation	0.750 (0.409-0.929)	0.653 (0.513-0.771)	0.668 (0.537-0.775)	0.860	0.681 (0.435-0.901)
BER-CNN	Training	0.922 (0.815-0.969)	0.914 (0.869-0.944)	0.915 (0.876-0.943)	0.812	0.976 (0.956-0.990)
	Internal Validation	0.938 (0.717-0.989)	0.455 (0.362-0.552)	0.521 (0.431-0.610)	0.863	0.660 (0.545-0.784)
	External Validation	0.500 (0.215-0.785)	0.898 (0.782-0.956)	0.842 (0.726-0.915)	0.860	0.628 (0.372-0.869)
BERT-DPCNN	Training	0.431 (0.305-0.567)	0.782 (0.723-0.831)	0.716 (0.659-0.766)	0.812	0.609 (0.524-0.692)
	Internal Validation	0.437 (0.231-0.668)	0.713 (0.618-0.792)	0.675 (0.586-0.753)	0.863	0.529 (0.354-0.695)
	External Validation	0.500 (0.215-0.785)	0.735 (0.597-0.838)	0.702 (0.573-0.805)	0.860	0.500 (0.254-0.736)
BERT-RNN	Training	0.765 (0.632-0.860)	0.577 (0.511-0.641)	0.613 (0.553-0.669)	0.812	0.702 (0.624-0.774)
	Internal Validation	0.750 (5050-8982)	0.574 (0.477-0.666)	0.598 (0.508-0.683)	0.863	0.643 (0.515-0.760)
	External Validation	0.625 (0.306-0.863)	0.816 (0.686-0.900)	0.789 (0.667-0.875)	0.860	0.640 (0.342-0.906)
BERT-RCNN	Training	0.784 (0.654-0.875)	0.450 (0.386-0.516)	0.513 (0.454-0.572)	0.812	0.624 (0.535-0.705)
	Internal Validation	0.812 (0.570-0.934)	0.356 (0.267-0.454)	0.419 (0.333-0.509)	0.863	0.569 (0.418-0.724)
	External Validation	0.875 (0.529-0.977)	0.347 (0.229-0.487)	0.421 (0.302-0.550)	0.860	0.523 (0.321-0.722)
Clinical Model	Training	0.333 (0.204-0.468)	0.873 (0.825-0.916)	0.771 (0.720-0.819)	0.812	0.582 (0.485-0.674)
	Internal Validation	0.312 (0.083-0.545)	0.861 (0.798-0.926)	0.786 (0.709-0.863)	0.863	0.641 (0.516-0.764)
	External Validation	0.111 (0.000-0.375)	0.880 (0.805-0.942)	0.804 (0.717-0.880)	0.902	0.675 (0.525-0.818)

Abbreviations: ACC, accuracy; AUC, area under the curve; CI, confidence interval; NIR, no information rate (proportion of the majority class).

Table S3 Performance of Multi-Scale and Multi-Modal Models in predicting recurrence

Model	Dataset	Sensitivity (95% CI)	Specificity (95% CI)	ACC (95% CI)	NIR	AUC (95% CI)
MSM	Training	0.980 (0.897-0.997)	0.827 (0.772-0.872)	0.856 (0.809-0.893)	0.812	0.944 (0.917-0.967)
	Internal Validation	0.875 (0.640-0.965)	0.733 (0.639-0.809)	0.752 (0.667-0.822)	0.863	0.837 (0.735-0.925)
	External Validation	0.625 (0.306-0.863)	0.837 (0.710-0.915)	0.807 (0.687-0.889)	0.860	0.681 (0.367-0.915)
MSM-T	Training	0.902 (0.790-0.957)	0.768 (0.708-0.819)	0.793 (0.741-0.837)	0.812	0.902 (0.864-0.939)
	Internal Validation	1.000 (0.806-1.000)	0.683 (0.587-0.766)	0.727 (0.639-0.799)	0.863	0.860 (0.786-0.921)
	External Validation	0.625 (0.306-0.863)	0.857 (0.733-0.929)	0.825 (0.706-0.902)	0.860	0.742 (0.549-0.913)
MSM-TC	Training	0.804 (0.675-0.890)	0.932 (0.891-0.958)	0.908 (0.867-0.937)	0.812	0.930 (0.887-0.965)
	Internal Validation	0.938 (0.717-0.989)	0.703 (0.608-0.783)	0.735 (0.649-0.807)	0.863	0.860 (0.780-0.925)
	External Validation	0.750 (0.409-0.928)	0.776 (0.641-0.870)	0.772 (0.648-0.862)	0.860	0.798 (0.644-0.914)

Abbreviations: MSM, Multi-Scale Model; MSM-T, Multi-Scale Model with Text; MSM-TC, Multi-Scale Model with Text and Clinical data; NIR, no information rate (proportion of the majority class).

Adjuvant treatments

Adjuvant chemotherapy was initiated within 2–3 weeks after surgery and continued every week for a total of 4–6 cycles with cisplatin (40 mg/m2) or carboplatin (AUC 5). Postoperative radiotherapy was performed within 3 weeks of radical surgery alone or initiated concurrently at the second and third cycle of chemotherapy. Whole-pelvic irradiation was given with 45 Gy-50 Gy in 1.8 Gy-2 Gy/ fraction by using the intensity modulated radiation therapy (IMRT) or volume intensity modulated radiotherapy (VMAT). Patients with vaginal invasion close to the surgical margin also received brachytherapy boost (10–14 Gy).

Preprocessing and Training Methodology

To prepare the dataset for model training, all T2-weighted MR images and their corresponding segmentation masks were first resampled to an isotropic voxel spacing of 1.0 × 1.0 × 1.0 mm^3 using linear and nearest-neighbor interpolation, respectively. Subsequently, dynamic region of interest (ROI) cropping was applied by localizing the tumor extent along the Z-axis to extract fixed-size consecutive slice blocks, alongside center-cropping the X-Y plane to a predefined patch size (32 × 32) based on the lesion's bounding box. Data augmentation methods, such as random rotation and random cropping were used during the training process.

All deep learning networks were trained end-to-end using the Adam optimizer with an initial learning rate of 0.01, whereas the parameters of the pretrained BERT text encoder were frozen throughout training. A stepwise learning-rate schedule was adopted, in which the learning rate was multiplied by 0.8 every 10 epochs. The model was trained for 200 epochs with a batch size of 8. Training was implemented using PyTorch 1.10.0 and CUDA 11.3 on a hardware platform equipped with a 12-core 3.30-GHz Intel Xeon Silver 4310 CPU and an NVIDIA GeForce RTX 3090 GPU. For the binary prediction task, focal loss with a weighting factor of $\alpha = 0.25$ and a focusing parameter of $\gamma = 2$ was applied directly to the raw prediction logits.

Model architecture

1 Overview

Given a single-channel three-dimensional region-of-interest (ROI) volume $\mathbf{X} \in \mathbb{R}^{B\times C_{\text{in}}\times D\times H\times W}$, the proposed imaging branch integrates dual-scale patch representations with hierarchical convolutional features to capture complementary volumetric information. As illustrated in Fig. [X], the overall framework comprises five sequential stages: (1) dual-scale patch embedding, which represents the input at complementary spatial granularities; (2) hierarchical query construction using a 3D ConvNeXt backbone; (3) query-context interaction through multi-head cross-attention; (4) sequence-level aggregation using a learnable classification token and a Transformer encoder; and (5) multimodal feature fusion and prediction. In the final stage, the aggregated imaging representation is combined with the

pooled textual representation and structured clinical variables to form a joint patient-level representation, which is subsequently normalized and mapped to a scalar prediction logit.

This design enables the network to preserve localized volumetric patterns, exploit multilevel convolutional context, and explicitly model interactions between the hierarchical imaging features and dual-scale patch representations before multimodal integration. In this study, $C_{\text{in}} = 1$ and $D = H = W = 32$. Other implementation-specific dimensions are summarized in Table 1 to avoid repeatedly embedding fixed numerical values in the mathematical formulation.

2 Dual-scale patch representation

To capture complementary anatomical information at different spatial granularities, the input volume is processed by two parallel patch-embedding branches with cubic patch sizes p_1 and p_2. The coarse branch captures broader volumetric context, whereas the fine branch preserves more localized structural details. For scale $i \in \{1,2\}$, the input volume is partitioned into non-overlapping cubic patches, and the corresponding number of patch tokens is

$$N_i = \left(\frac{D}{p_i}\right)\left(\frac{H}{p_i}\right)\left(\frac{W}{p_i}\right)$$

Each flattened patch is linearly projected into a shared d-dimensional embedding space, yielding the scale-specific token sequence $\mathbf{V}_i \in \mathbb{R}^{B \times N_i \times d}$. The two sequences are then concatenated along the token dimension to construct a unified multiscale context representation:

$$\mathbf{C} = \text{Concat}_{\text{token}}(\mathbf{V}_1, \mathbf{V}_2) \in \mathbb{R}^{B \times N \times d}, \qquad N = N_1 + N_2$$

In the implemented model, $p_1 = 16$, $p_2 = 8$, and $d = 256$, resulting in 8 coarse-scale tokens and 64 fine-scale tokens. These values represent implementation choices rather than intrinsic constraints of the proposed formulation.

3 Hierarchical query construction using 3D ConvNeXt

In parallel with the patch-embedding branches, the input volume is processed by a 3D ConvNeXt backbone to extract hierarchical convolutional representations. Each block comprises a depthwise $7 \times 7 \times 7$ convolution, normalization, two pointwise transformations separated by a Gaussian error linear unit (GELU), layer scaling, and a residual connection. Because these operations follow the conventional ConvNeXt formulation, they are described textually rather than restated using an additional equation.

Let $\mathbf{F}_l \in \mathbb{R}^{B\times C_l\times D_l\times H_l\times W_l}$ denote the feature map extracted from the lth selected stage. A stage-specific projection $\phi_l(\cdot)$ aligns the channel dimensions to a common width C_q. The projected feature maps are flattened along their spatial dimensions and concatenated to construct the hierarchical query representation:

$$\mathbf{H} = \mathrm{Concat}_{\mathrm{spatial}}(\mathrm{Flatten}[\phi_1(\mathbf{F}_1)], \dots, \mathrm{Flatten}[\phi_L(\mathbf{F}_L)]) \in \mathbb{R}^{B\times C_q\times M}, \qquad M = \sum_{l=1}^{L} D_l H_l W_l$$

Importantly, the channel dimension is treated as the query-token axis, whereas the concatenated spatial responses across multiple resolutions constitute the descriptor dimension. This channel-indexed tokenization follows the implemented reshape-and-concatenation procedure and differs from the conventional strategy in which individual spatial positions are regarded as tokens. Three ConvNeXt stages are used in the present implementation. Their spatial resolutions are 8^3, 4^3, and 2^3, respectively; after channel alignment, $C_q = 96$ and $M = 584$.

4 Cross-attention-based multiscale fusion

The hierarchical representation $\mathbf{H}$ serves as the query sequence, whereas the dual-scale patch representation $\mathbf{C}$ provides the key and value sequences. This asymmetric interaction enables each channel-indexed query token to adaptively retrieve complementary contextual information from both coarse- and fine-scale patch tokens. For each attention head, the projected queries, keys, and values are denoted by $\mathbf{Q}$, $\mathbf{K}$, and $\mathbf{V}$, respectively. The scaled dot-product attention is defined as

$$\mathrm{Attn}(\mathbf{Q},\mathbf{K},\mathbf{V}) = \mathrm{softmax}\left(\frac{\mathbf{Q}\mathbf{K}^{\mathrm{T}}}{\sqrt{d_h}}\right)\mathbf{V}$$

where d_h denotes the dimensionality of each attention head. The head-wise outputs are concatenated and linearly projected to the query-embedding space, yielding

$$\mathbf{W} = \mathrm{MHA}(\mathbf{H},\mathbf{C},\mathbf{C})$$

The fused representation $\mathbf{W}$ retains the channel-indexed query structure while incorporating a data-dependent combination of the dual-scale contextual features. The softmax operation is evaluated over the context-token dimension, and no attention mask is applied. The implemented cross-attention module contains eight heads, each with a head dimension of 64.

5 Classification-token-based sequence aggregation

To obtain a fixed-dimensional imaging representation, a learnable classification token $\mathbf{c}_{\text{cls}}$ is prepended to the fused query sequence, and a learnable positional embedding $\mathbf{P}$ is added. The resulting sequence is processed by a pre-normalized Transformer encoder:

$$\mathbf{S}_0 = [\mathbf{c}_{\text{cls}}; \mathbf{W}] + \mathbf{P}, \qquad \mathbf{v} = \text{Transformer}(\mathbf{S}_0)[:,0,:]$$

The output associated with the classification token, $\mathbf{v}$, summarizes the interactions among the fused hierarchical query tokens and serves as the final imaging representation. The Transformer encoder comprises 12 layers, each containing multi-head self-attention and a GELU-activated feed-forward network. Dropout is set to 0.1. The self-attention module employs six heads with explicit head-wise projections, followed by an output projection to the sequence-embedding dimension.

6 Multimodal feature fusion and prediction

The accompanying textual information is encoded using a frozen pretrained BERT encoder, whose pooled output is retained as the textual representation $\mathbf{t} \in \mathbb{R}^{B\times d_t}$. The Transformer-aggregated imaging representation is denoted by $\mathbf{v} \in \mathbb{R}^{B\times d_v}$, and the structured clinical variables are represented by $\mathbf{z} \in \mathbb{R}^{B\times d_z}$. Rather than combining independently generated modality-specific decisions, the proposed framework performs feature-level late fusion, thereby allowing the final predictor to jointly consider semantic information from the clinical text, volumetric imaging characteristics, and structured patient-level covariates.

The three modality-specific representations are concatenated along the feature dimension to form the joint multimodal representation

$$\mathbf{f} = \text{Concat}_{\text{feature}}(\mathbf{t}, \mathbf{v}, \mathbf{z}) \in \mathbb{R}^{B\times d_f}, \qquad d_f = d_t + d_v + d_z$$

Because the constituent representations originate from heterogeneous encoders and may exhibit different feature distributions, layer normalization is applied to the fused vector before prediction. The normalized multimodal representation is then mapped to a scalar logit through a learnable linear decision function:

$$\tilde{\mathbf{f}} = \text{LN}(\mathbf{f}), \qquad \mathbf{s} = \tilde{\mathbf{f}}\mathbf{w}_o + b_o$$

where $\mathbf{w}_o \in \mathbb{R}^{d_f\times 1}$ and $b_o \in \mathbb{R}$ denote the learnable classifier parameters. The resulting $\mathbf{s} \in \mathbb{R}^{B\times 1}$ contains one unbounded prediction logit for each patient. For binary classification, the

corresponding probability is obtained as

$$\hat{\mathbf{p}} = \sigma(\mathbf{s})$$

where $\sigma(\cdot)$ denotes the logistic sigmoid function. During model optimization, the raw logits are supplied directly to a logits-based binary classification objective, whereas the sigmoid transformation is applied during inference to obtain interpretable probabilities. A prespecified decision threshold may subsequently be used to convert these probabilities into binary predictions.

In the implemented model, $d_t = 768$, $d_v = 584$, and $d_z = 5$, resulting in a fused representation with $d_f = 1357$. The definitions, encoding procedures, normalization strategies, and missing-value handling methods for the five structured clinical variables are described in the data preprocessing subsection.
